# γ-Conglutin Immunoreactivity Is Differently Affected by Thermal Treatment and Gastrointestinal Digestion in Lupine Species

**DOI:** 10.3390/foods13152330

**Published:** 2024-07-24

**Authors:** Caterina Villa, Bruno Carriço-Sá, Carla S. S. Teixeira, Catarina Dias, Renata Costa, Carlos M. Pereira, Isabel Mafra, Joana Costa

**Affiliations:** 1 REQUIMTE-LAQV, Faculdade de Farmácia, Universidade do Porto, Rua de Jorge Viterbo Ferreira, 228, 4050-313 Porto, Portugal; up201805289@edu.fc.up.pt (B.C.-S.); cteixeira@ff.up.pt (C.S.S.T.); up201606196@edu.fc.up.pt (C.D.); isabel.mafra@ff.up.pt (I.M.); jbcosta@ff.up.pt (J.C.); 2 Instituto de Ciências Moleculares/Centro de Investigação em Química da Universidade do Porto (IMS/CIQUP), Faculdade de Ciências da Universidade do Porto, Departamento de Química e Bioquímica, Rua do Campo Alegre 687, 4169-007 Porto, Portugal; renata.costa@fc.up.pt (R.C.); cmpereir@fc.up.pt (C.M.P.)

**Keywords:** food allergens, lupine, IgG binding, food products, thermal treatment, gastrointestinal digestion

## Abstract

Lupine is a legume commonly used in human diet as a functional food due to its high nutritional content and important technological properties. However, its consumption can lead to the manifestation of adverse immunological reactions, posing significant health issues in sensitized/allergic patients. This work aims to investigate the effect of food processing combined with simulated gastrointestinal (GI) digestion on the immunoreactivity of lupine γ-conglutin. Model foods of wheat pasta containing 35% of lupine flour (*Lupinus albus*, *L. luteus*, and *L. angustifolius*) were prepared and submitted to a boiling process. The proteins were extracted and their profiles characterized by SDS-PAGE. Simulated GI digestion was performed on thermally treated pasta using the INFOGEST harmonized digestion protocol 2.0. The IgG binding capacity of γ-conglutin was assessed by immunoblotting in non-reducing conditions and indirect ELISA with specific antibodies. Results demonstrate that the boiling treatment affected the immunoreactivity of the three lupine species differently. Simulated GI digestion led to extensive destruction of the protein structure, more significant in the intestinal phase, reducing but not abolishing the IgG affinity to γ-conglutin and its potential presentation to immunocompetent cells. This information can offer valuable insights to the food industry for developing food formulations with reduced allergenic properties.

## 1. Introduction

Lupine has gained particular attention as a technological ingredient for food and beverage industry due to its nutritional value and functional properties, as well as its ability to adapt to harsh conditions and low-input farming. The excellent functional properties of lupine flour, such as water absorption, emulsifying capacity, and foam stability, make it a versatile ingredient in food manufacturing, commonly used to improve the texture, moisture retention, and shelf life of foodstuffs [1,2]. In fact, lupine is rich in protein, dietary fiber, vitamins, and minerals [1], presenting high content in essential amino acids, which makes it a complete protein source. Additionally, lupine flour is often used as a gluten-free alternative to produce bread, cakes, and other baked goods for individuals with celiac disease or gluten sensitivity [2]. According to FAOSTAT, the production of lupine increased from 1.1 million tons in 2020 to 1.6 in 2022, accounting for 12 million dollars in export value [3]. The expanding use of lupine as a technological ingredient and healthy food in human nutrition has raised the concern about its allergenic potential. In fact, lupine has emerged as a common allergenic food, especially in countries where it is largely consumed, such as Mediterranean countries and Australia, with a sensitization prevalence of around 0.3–8% in Europe [1,4,5]. Since the primary case of an allergic reaction after the ingestion of a lupine-fortified pasta in 1994 reported by Hefle, et al. [6], the number of cases has substantially increased over the years. Currently, lupine and lupine-based products are included in a list of 14 foods or substances likely to cause allergies or intolerances, with the European Union determining their mandatory inclusion in pre-packaged foodstuffs labeling [7].

The severity of symptoms in patients suffering from lupine allergy can range from vomiting and urticaria to anaphylaxis, owing to the ingestion of allergenic proteins [8]. The protein amount among different lupine species can vary from 28% to 48%, approximately doubling the protein content found in other legumes commonly consumed by humans, such as lentils, peas, or soybeans [8]. The main allergenic protein classes identified in lupine are the globulins (α-, β-, and γ-conglutin) and 2S albumins (δ-conglutin), with some minor fractions, including pathogenesis-related (PR)-10 proteins, nonspecific lipid transfer proteins (nsLTP), and profilins [9,10]. Magni et al. [11] recognized γ-conglutin as one of the most allergenic proteins in lupine due to its specific structure and composition. This protein is a basic 7S globulin of 46 kDa consisting of two subunits, 29 and 17 kDa, respectively, linked together with a single disulfide bond and accounting for 5% of the total protein in *Lupinus albus* [11,12,13].

Since lupine is used in a wide range of processed foods, efforts have been made to find strategies to diminish the risk associated with its ingestion by lupine allergic patients. The use of common and/or novel food processing techniques has demonstrated interesting results in modulating lupine allergenicity by shaping their protein physicochemical properties [8,14]. Actually, food processing can lead to structural and chemical modifications in lupine proteins, which can alter their allergenicity by changing some epitopes or exposing new ones [15]. Accordingly, Villa, Moura, Costa, and Mafra [5] observed a decrease in lupine immunoreactivity after applying a baking treatment (180 °C) in lupine-fortified breads, with substantial alterations on the integrity and structure of γ-conglutin, depending on the lupine species [5]. In parallel, the effect of gastrointestinal (GI) digestion needs also to be considered. Some allergenic proteins can resist to the severe conditions of digestion even after food processing, allowing for them to be absorbed by the intestinal mucosa and became accessible to immunocompetent cells, thus increasing their potential for sensitization [16]. A recent study developed by Czubiński, et al. [17] demonstrated that lupine globulins are differently affected by digestive enzymes, with γ-conglutin being more susceptible to gastric enzymes than to intestinal ones, such as trypsin and pancreatin.

*L. albus*, *L. luteus*, and *L. angustifolius*, from the Leguminosae family, are the main lupine species used in human nutrition, all being considered potentially allergenic to lupine-allergic patients [8]. Following the current tendency to find strategies to reduce the risk associated with lupine ingestion, it is important to understand how food processing combined with GI digestion can influence the structural features of lupine proteins and their consequent effects on immunoreactivity. Thereby, studies focusing on this type of protein modification are essential to advance knowledge towards the development of lupine-containing products with reduced allergenicity. In this work, we investigate the use of boiling as thermal processing combined with GI digestion for a potential reduction in γ-conglutin immunoreactivity of the three most economically important species of lupine. For this purpose, model mixtures of homemade pasta were prepared, each containing one of the three economically important lupine species and submitted to boiling treatment, followed by *in vitro* GI digestion. The combined effect of thermal treatment and GI digestion on the immunoreactivity of γ-conglutin of each lupine species was evaluated by immunochemical assays.

## 2. Materials and Methods

### 2.1. Model Mixtures Preparation

*L. albus* and *L. luteus* flours were furnished by the company Germisem (Coimbra, Portugal), while the flour of *L. angustifolius* was acquired at a local market. Three independent sets of model mixtures with 35.0% (*w*/*w*) of each lupine flour (*L. albus*, *L. luteus*, or *L. angustifolius*) were prepared, following the traditional recipe for homemade pasta preparation, employing wheat flour (type 65), eggs (size M), olive oil, and salt as ingredients. After mixing lupine and wheat flours to obtain the required proportion, the flour mixture was shaped into a typical volcano shape in which the eggs were cracked and placed in the center, together with olive oil and salt. The obtained mixture was worked for 15 min until the formation of a consistent and elastic dough ball, which was left to rest for at least 30 min at 4 °C. Each model mixture was divided into two parts: one used as raw sample and the other to be submitted to a boiling process during 5 min to simulate the common processing of homemade pasta. Before processing, the dough was smashed and cut into rectangular strips to reproduce the shape of commercialized pasta (tagliatelle). After the boiling process a small quantity of boiling water was collected. The boiled pasta was dried during several minutes and then minced/homogenized in a laboratory knife mill (Grindomix GM200, Retsch, Haan, Germany). Both raw and processed samples were preserved at −20 °C until further analysis.

Different food species (10) described to be cross-reactive with lupine were tested for antibody specificity, including milk, tree nuts (hazelnut and walnut), legumes (peanut, fava bean, bean, chickpea, pea, and lentil), and wheat [5].

### 2.2. Protein Extraction and Quantification

Each raw and boiled model mixture of the homemade pasta was submitted to a protein extraction protocol performed by adding 1.5 mL of Tris–HCl buffer (100 mM, pH 8.0) to each pasta sample (150 mg), followed by an incubation at 60 °C for 2 h with constant agitation at 950 rpm. The mixtures were then centrifuged twice at room temperature (9000× *g*, 30 min) with the collection of supernatant between centrifugations. After extraction, protein concentration was measured by UV spectrophotometry (SPECTROstar Nano, BMG Labtech, Ortenberg, Germany) using an LVis plate accessory of reduced volume (BMG Labtech, Ortenberg, Germany). The protein content was assessed by the protein quantification protocol and the absorbance data were evaluated with MARS data analysis software (BMG Labtech, Ortenberg, Germany).

### 2.3. SDS-PAGE Analysis

Sodium dodecyl sulfate–polyacrylamide gel electrophoresis (SDS-PAGE) (5–12% or 5–15%) gels in discontinuous system were homemade, as described in the Mini-PROTEAN^®^ Tetra Cell Instruction Manual [18]. SDS-PAGE analysis allows for assessing the protein profile of each model mixture and boiling water. Therefore, all protein extracts obtained from model mixtures of raw and boiled homemade pasta, as well as all boiling water, were run at 150 V in non-reducing conditions by adding 2× Laemmli Sample Buffer (Bio-Rad Laboratories, Inc., Hercules, CA, USA) to each extract/boiled water in a 1:1 ratio (10 μL of each). A Mini-PROTEAN^®^ Tetra System (Bio-Rad Laboratories, Inc., Hercules, CA, USA) was used, with 1× Tris/Glycine/SDS (Bio-Rad Laboratories, Inc., Hercules, CA, USA) as an electrophoresis buffer. Proteins were diluted in the range of 1 to 15 µg, according to the total protein quantity obtained by UV spectrophotometry, as explained in Section 2.2, and loaded into gels. The proteins were visualized by staining the gels with Coomassie Brilliant Blue G-250 solution or blotted into a nitrocellulose membrane for subsequent immunoblot analysis. Gel image was collected using a white tray and processed with Image Lab 5.2.1 software (Gel Doc^™^ EZ Imager, Bio-Rad Laboratories, Inc., Hercules, CA, USA). Precision Plus Protein^™^ Dual Xtra Prestained Protein Standards (10–250 kDa, Bio-Rad Laboratories, Inc., Hercules, CA, USA) was used as protein molecular weight reference.

### 2.4. Immunoblotting Analysis

The effect of boiling treatment followed by GI digestion on the immunoreactivity of γ-conglutin was evaluated by immunoblotting analysis. Therefore, after electrophoresis, proteins were transferred by blotting the gels into a nitrocellulose membrane using a Trans-Blot^®^ Turbo^™^ Transfer System (Bio-Rad Laboratories, Inc., Hercules, CA, USA) with an automatic turbo protocol (2.5 A, up to 25 V, 7 min), following the Trans-Blot^®^ Turbo^™^ Transfer System Instruction Manual [19]. The membranes were then washed three times with 30 mL of TBST 1× (pH 7.4, 10 mM Tris, 50 mM NaCl, 0.1% Tween 20) for 10 min. A blocking step was performed with 30 mL of TBST 1× containing 2% of gelatin from cold-water fish skin (Sigma-Aldrich, St Louis, MO, USA) for 1 h at room temperature, and washed again 3 times with TBST 1× (10 min each). The membranes were then incubated overnight at 4 °C with a primary antibody specific to lupine (rabbit anti-lupine γ-conglutin, Agrisera, Sweden) diluted 1/50,000 in incubation buffer (0.6 μL of primary antibody in 30 mL of TBST 1× with 2% fish gelatin). A second incubation was performed with a secondary antibody (anti-rabbit IgG combined with peroxidase, produced in goat (Sigma-Aldrich, St Louis, MO, USA)), diluted 1/40,000 in incubation buffer (0.75 μL of secondary antibody in 30 mL of TBST 1× with 2% fish gelatin), for 2 h, at room temperature. Between incubations, the membranes were washed again in the same conditions. The membranes were revealed with Clarity™ Western ECL (Bio-Rad Laboratories, Hercules, CA, USA), and visualized using the ChemiDoc^TM^ Touch Imaging System and processed with Image Lab 5.2.1 software (Gel Doc^TM^ EZ Imager, Bio-Rad Laboratories, Inc., Hercules, CA, USA).

### 2.5. Indirect ELISA

An indirect ELISA was executed to detect γ-conglutin in protein extracts from homemade pasta/boiling water and in gastric and intestinal digested samples, and in this way, quantify the effective increase/reduction in immunoreactivity. High binding capacity polystyrene 96-well plates, namely, Nunc MaxiSorp™, were used. Different calibration curves were assayed to determine the best dynamic range for the detection of γ-conglutin in each lupine species (*L. albus*, *L. luteus*, or *L. angustifolius*): 1–1000 pg/mL and 5–200 pg/mL (8 concentration levels per curve). Additionally, calibration curves using the combination of all target species were also tested in the range of 0.5–5 μg/mL, 0.5–10 μg/mL, 0.5–15 μg/mL, and 0.5–25 μg/mL (*n* = 8 standards per calibration curve). Briefly, 100 μL of protein extracts, digests, or calibration curve solutions diluted in coating buffer (carbonate/bicarbonate/azide buffer (100 mM; pH 9.4)) in order to obtain a signal within the calibration curve were incubated for 2 h. The plates were then washed 3 times with 200 μL of 1× PBST (10 mM PBS (pH 7.5, 0.1% Tween 20)), and blocked with 200 μL of 2% of fish gelatine in coating buffer (carbonate/bicarbonate/azide buffer 100 mM, pH 9.4) at room temperature for 2 h. After washing (3 times) with 200 μL of 1× PBST, the plates were incubated overnight at 4 °C with 100 μL of anti-γ-conglutin antibody diluted 1/40,000-fold in assay buffer (50 mM PBS, 0.1% Tween 20, 2% of fish gelatine). A washing step was performed again (3 times with 200 μL of 1× PBST), followed by a second incubation for 2 h at room temperature with 100 μL of anti-rabbit IgG peroxidase antibody 40,000-fold diluted in assay buffer. At the end of the final washing steps (3 times with 200 μL of 1× PBST), the plates were incubated with 50 μL of tetramethylbenzidine (TMB) for approximately 5 min in the dark, until the development of a blue coloration indicative of product formation. The reaction was stopped with 50 μL of 2 M H_2_SO_4_, leading to the formation of a yellow color in the wells. The ELISA plates were read at 450 nm in a plate reader (SPECTROstar Nano, BMG Labtech, Ortenberg, Germany) and the absorbance data were evaluated with MARS data analysis software (BMG Labtech, Ortenberg, Germany). For data analysis, the absorbance values measured at 450 nm were plotted against the concentration of γ-conglutin in each species or in the combination of all species, using a non-linear regression function (4-parametric logistic function):

$$Y=\frac{A-D}{1+{\left(\frac{X}{C}\right)}^{b}}+D$$

where Y is the optical density (absorbance), *A* is the maximum absorbance, *b* is the slope of the calibration curve in the linear range, *C* is the 50% inhibition concentration (IC50, μg/L), *D* is the minimum absorbance, and *X* is the analyte concentration (μg/L). The obtained results are expressed as mg/mL of γ-conglutin. Each concentration from the calibration curve and protein extracts/digests were analyzed in *n* = 4 replicates and repeated in 3 independent assays (on 3 different days).

### 2.6. Gastrointestinal Digestion

The *in vitro* GI digestion were performed following a standardized static digestion protocol, simulating oral, gastric, and intestinal phases, as reported by Brodkorb et al. [20]. Simulated salivary fluid (SSF, pH 7) contained 15.1 mM KCl, 3.7 mM KH_2_PO_4_, 13.6 mM NaHCO_3_, 0.15 mM MgCl_2_, 0.06 mM (NH_4_)_2_CO_3_, and 1.1 mM HCl for pH adjustment. Simulated gastric fluid (SGF, pH 3) was prepared with 6.9 mM KCl, 0.9 mM KH_2_PO_4_, 25 mM NaHCO_3_, 47.2 mM NaCl, 0.12 mM MgCl_2_, 0.5 mM (NH_4_)_2_CO_3_, and 15.6 mM HCl for pH adjustment. Simulated intestinal fluid (SIF, pH 7) was prepared with the addition of 6.8 mM KCl, 0.8 mM KH_2_PO_4_, 85 mM NaHCO_3_, 38.4 mM NaCl, 0.33 mM MgCl_2_, and 8.4 mM HCl for pH adjustment. All simulated fluids were prepared according to a final volume of 400 mL. As precipitation may occur, CaCl_2_(H_2_O)_2_ (0.3 M) solution was not added to the electrolyte stock solutions, but rather added at the same time of each stock fluid.

Each raw and boiled pasta containing 35% of lupine flour were submitted to *in vitro* GI digestion according to the standardized protocol described by Brodkorb et al. [20]. All steps were performed in a shaking incubator (37 °C, 170 rpm). To assess protein degradation along the digestion process, individual sample tubes were used to analyze digests in different time points. Oral phase was performed by adding 400 µL of SSF, 3 µL of 0.3 M CaCl_2_ solution, and 97 µL of ultrapure H_2_O to 500 mg of each model pasta to complete 500 mL of total salivary mixture (bolus). The mixture was mashed using a glass rod for 30 s and incubated for a total of 2 min. Afterward, the chewed mixture was mixed in a 1:1 proportion with SGF and 1 µL of 0.3 M CaCl_2_ solution containing 2000 U/mL of gastric pepsin (Sigma-Aldrich, St. Louis, MO, USA) to simulate gastric digestion. The pH was adjusted to 3.0 with 6M HCl solution and a volume of 2 mL was completed by adding ultrapure H_2_O. Mixtures were then incubated at 37 °C for 2 h. To stop gastric digestion 1 M NaOH solution was added followed by immediate storage in ice. Intestinal phase was carried out at 37 °C for 2 h by incorporating SIF (1:1 *w*/*v*), 4 µL of 0.3 M CaCl_2_, and 10 mM of bile salts (Sigma-Aldrich, St. Louis, Missouri, USA) with pancreatin (100 U/mL) or individual enzymes (trypsin (100 U/mL) and chymotrypsin (25 U/mL)). The pH was adjusted to 7.0 with 1 M NaOH solution and a volume of 4 mL was completed by the addition of ultrapure water. Phenyl methane sulfonyl fluoride (PMSF) 0.1 M was used to stop the intestinal enzymatic activity. All time points were performed in independent tubes simulating the GI digestion process. All tubes were centrifuged at 9000× *g* for 15 min (4 °C) and the supernatant was collected and stored at −20 °C until further analysis. The total protein concentration was calculated by UV spectrophotometry according to Section 2.2.

## 3. Results

In this study, the effect of heat processing combined with GI digestion on the IgG binding capacity of γ-conglutin was evaluated in different species of lupine, namely, *L. albus, L. luteus*, and *L. angustifolius.* For that purpose and to mimic processed food matrices containing lupine as an ingredient as much as possible, model mixtures simulating the homemade pasta were prepared with 35% of lupine flour, and submitted to a typical boiling process for 5 min. The protein profile and IgG binding capacity of γ-conglutin, before and after the application of the thermal treatment, were evaluated by SDS-PAGE and immunoblotting, respectively. To evaluate the influence of the GI digestion on the protein profile, the INFOGEST 2.0 protocol was performed using pepsin in the gastric phase and pancreatin or individual enzymes (trypsin and chymotrypsin) in intestinal phase [20]. An indirect ELISA was finally executed to quantify the changes (loss or increase) on IgG binding signal after processing and GI digestion of the pasta mixtures.

### 3.1. Effect of Thermal Treatment

#### 3.1.1. SDS-PAGE and Immunoblotting with Polyclonal Antibodies

**Figure 1:**
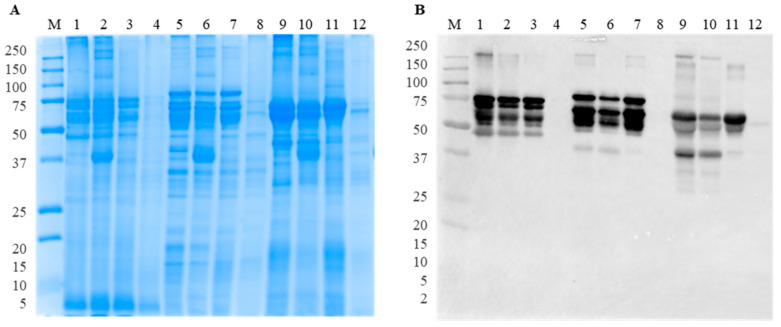
Protein profile and IgG binding capacity of γ-conglutin by SDS-PAGE in non-reducing conditions (**A**) and immunoblot membranes using anti-γ-conglutin polyclonal antibody (**B**). Legend: lane M, Precision Plus Protein Dual Xtra Prestained Protein Standards 2–250 kDa (Bio-Rad Laboratories, Inc., Hercules, CA, USA); lane 1, *L. luteus* flour; lane 2, raw pasta containing 35% of *L. luteus*; lane 3, boiled pasta containing 35% of *L. luteus*; lane 4, water from *L. luteus* boiling pasta; lane 5, *L. angustifolius* flour; lane 6, raw pasta containing 35% of *L. angustifolius*; lane 7, boiled pasta containing 35% of *L. angustifolius*; lane 8, water from *L. angustifolius* boiling pasta; lane 9, *L. albus* flour; lane 10, raw pasta containing 35% of *L. albus*; lane 11, boiled pasta containing 35% of *L. albus*; lane 12, water from *L. albus* boiling pasta.

Differences in the protein profile and IgG binding of raw lupine seeds from the three of the most economically important lupine species were already evaluated in a previous study, with interesting results [5]. In fact, it was suggested that the distinct patterns of immunoreactive bands among the lupine species could be correlated with the different degrees of protein glycosylation and the existence of subunits with different molecular weight according to the target species. For that reason, it is critical to understand whether these differences in immunoreactivity among species persist after food processing and GI digestion. Figure 1 shows the comparison results between protein profile and IgG binding pattern of each lupine species and pasta mixture, before and after processing, as well as the boiling water collected after thermal treatment. Bands at 250 kDa represent the native form of γ-conglutin, corresponding to a trimeric/hexameric quaternary structure, similar to most vicilin-like globulins [21]. In *L. luteus* (lanes 1–3, Figure 1B) and *L. angustifolius* (lanes 5–7, Figure 1B), the most immunoreactive bands have between 45 and 75 kDa, while in *L. albus*, bands are within 45–60 kDa (lanes 9–11, Figure 1B). Czubinski et al. [22] identified a single band at 44.2 kDa under non-reducing SDS-PAGE conditions as belonging to coupled α- (31.4 kDa) and β- (16.5 kDa) subunits of mature γ-conglutin [22]. The presence of bands with a slightly higher molecular weight can be due to N-linked glycosylation, a post-translational modification (PTM) associated with this protein, which may change its electrophoretic mobility [22,23], or the presence of γ-conglutin monomers and dimers [24]. An additional band at approximately 37 kDa is present in *L. angustifolius* (lanes 5 and 6, Figure 1B) and in *L. albus* (lanes 9 and 10, Figure 1B), which disappeared after the boiling treatment (lanes 7 and 11, Figure 1B). This band is possibly the glycosylated form of the α-subunit, presenting a higher molecular weight than the expected (31.4 kDa). Regarding the bands at 45–75 kDa, the immunoreactivity of γ-conglutin seems to be differently affected by the processing of each species. In fact, the intensity of bands remains almost unchanged after boiling in *L. luteus*, while in *L. angustifolius* and *L. albus*, they seem to increase, compared with their raw counterparts. Thermal treatment can lead to the denaturation of this target protein and, consequently, to the loss of some conformational epitopes or the formation of new ones, as it seems to happen in *L. angustifolius* and *L. albus*. Lastly, as observed in SDS-PAGE results, some proteins migrated to the boiling water (lanes 4, 8, and 12, Figure 1A), but none of them correspond to γ-conglutin (lanes 4, 8, and 12, Figure 1B).

#### 3.1.2. Indirect ELISA

As an attempt to quantify the change on the γ-conglutin immunoreactivity after the application of thermal treatment, previously suggested by immunoblotting analysis, an indirect ELISA using the same polyclonal antibody was performed. The results are summarized in Table 1. Comparing the signal from all raw pasta, substantial differences can be observed among the values of γ-conglutin obtained for each lupine species. Accordingly, a decrease of 2-fold was found between *L. luteus* and *L. angustifolius*, while *L. albus* presented a 100- and 50-fold less reactivity towards *L. luteus* and *L. angustifolius*, respectively. This fact can be attributed to the intrinsic specificity of the antibody (raised against *L. luteus* γ-conglutin), which is clearly more reactive to *L. luteus* and *L. angustifolius*, followed by *L. albus*, and confirms the presence of distinct immunoreactive epitopic regions of the protein in each species. The immunoreactivity of *L. luteus* presented a reduction of 40% after the boiling treatment (from 2.47 ± 0.11 to 1.50 ± 0.24 mg/mL); *L. angustifolius* maintained the same IgG binding rate (1.23 ± 0.14 and 1.22 ± 0.06 mg/mL), while *L. albus* had a significant increase above 5000% (from 0.0231 ± 0.0011 to 1.17 ± 0.10 mg/mL). These three different scenarios seem to be dependent on the lupine species, being caused by distinct factors. In the first case, the structural alteration of γ-conglutin in *L. luteus* led to the destruction of conformational epitopes, due to the boiling process, although preserving the high immunoreactivity of its linear epitopes. In the second case, the application of temperature did not affect the epitopic regions of γ-conglutin in *L. angustifolius* as both raw and processed pasta maintain the immunoreactivity unaltered. In the last case, the scenario was contradictory, considering that the immunoreactivity of this protein was probably limited to internal linear epitopes, as non-denatured γ-conglutin in *L. albus* renders low reactivity towards its specific antibody. All the collected boiling waters exhibited residual immunoreactivity in the case of *L. angustifolius* and *L. albus*, but more relevant for *L. luteus* (0.0004 ± 0.0001 to 0.22 ± 0.02 mg/mL), which is somewhat proportional to the differences observed between raw and boiled pasta.

In general, these results confirm the immunoblotting data and demonstrate that the IgG binding pattern of γ-conglutin is specific for each lupine species and is differently affected by food processing. Therefore, it can be assumed that the partial denaturation of the protein or the formation of aggregates induced by thermal treatment occur in distinct ways according to the lupine species. These modifications can lead to the disruption of conformational epitopes or the formation of new ones, with the consequent increase or reduction in allergenicity, respectively.

First reports studying the effect of thermal treatments on lupine indicate that lupine proteins are mainly heat-resistant after boiling and roasting [25,26,27]. Soft, medium, and harsh thermal treatments normally used in industrial processing were applied to *L. angustifolius* seeds with only partial or no effect on the stability of the released peptides [28]. Álvarez-Álvarez, Guillamón, Crespo, Cuadrado, Burbano, Rodríguez, Fernández, and Muzquiz [25] demonstrated that only autoclaving produced a relevant reduction of IgE binding in *L. albus*, affecting the integrity and structure of proteins. The boiling process (15, 30, and 60 min) seemed to have no effect on *L. albus* IgE binding, but data showed an increase in the band intensity in immunoblotting with sera from patients allergic to lupine, which suggested a correlation with the increase of γ-conglutin immunoreactivity from *L. albus* observed in this work. However, Villa, Moura, Costa, and Mafra [5] observed a general decrease in *L. albus* immunoreactivity after a baking treatment at 180 °C, while Holden et al. [29] reported a reduction in the IgE binding capacity of *L. albus* seeds in a tofu-like product (Lopino), prepared by soaking seeds, followed by the application of a boiling and pressure treatment. These distinct ways of transferring heat to the food by boiling (moist-heating) or by baking (dry-heating) may have an impact on how much energy reaches the proteins, inducing different modifications to their structure and potentially affecting the epitopes and their IgE binding capacity. All the mentioned studies refer to *L. albus* and one to *L. angustifolius,* without any study focusing on *L. luteus* or on the comparison of the effect of processing on the three economically important lupine species.

The N-linked glycosylation can also play an important role here since it seems that this type of PTM can affect the thermal stability of the protein [30], which in turn can affect its immunoreactivity. Homologue proteins from lupine seed species present different degrees of glycosylation, with the presence of significantly more heterogeneous and complex glycans in *L. angustifolius* γ-conglutin than in its *L. albus* homologue [30,31]. A specific class of asparagine-linked oligosaccharides attached to *L. albus* γ-conglutin and containing xylose and/or a core-linked α-1,3-fucose is considered a cross-reactive carbohydrate determining factor (CCD), which tends to interact with specific IgE in *in vitro* assays, although not eliciting clinical symptoms. The N-glycosylated proteins can give false positive results in allergy diagnosis [31], thus potentially explaining the observed findings. Understanding the role of glycosylation of γ-conglutin is crucial, as its structural properties and, consequently, its allergenicity could be potentially influenced by the type and complexity of bound carbohydrate according to each lupine species.

### 3.2. Effect of Gastrointestinal Digestion

After boiling pasta mixtures, it was possible to understand that heat treatment led to distinct structural and immunoreactive modifications on γ-conglutin according to lupine species. At this point, testing the combination of food processing with GI digestion became crucial to verifying its influence on lupine immunoreactivity. Therefore, GI digestion of boiled model lupine pasta was performed following the standardized protocol of INFOGEST 2.0 [20] with pancreatin or individual enzymes in the intestinal phase. Pancreatin is a mixture of several digestion enzymes in which trypsin, chymotrypsin, and lipases are the principal enzymes. Trypsin is a highly specific protease, showing specificity to hydrolyze peptide bonds, only between positively charged amino acids, and cleaves peptide chains generally at the carboxyl side of lysine or arginine, while chymotrypsin preferentially cleaves peptide chains on the carboxyl side of tyrosine, tryptophan, or phenylalanine [32].

**Figure 2:**
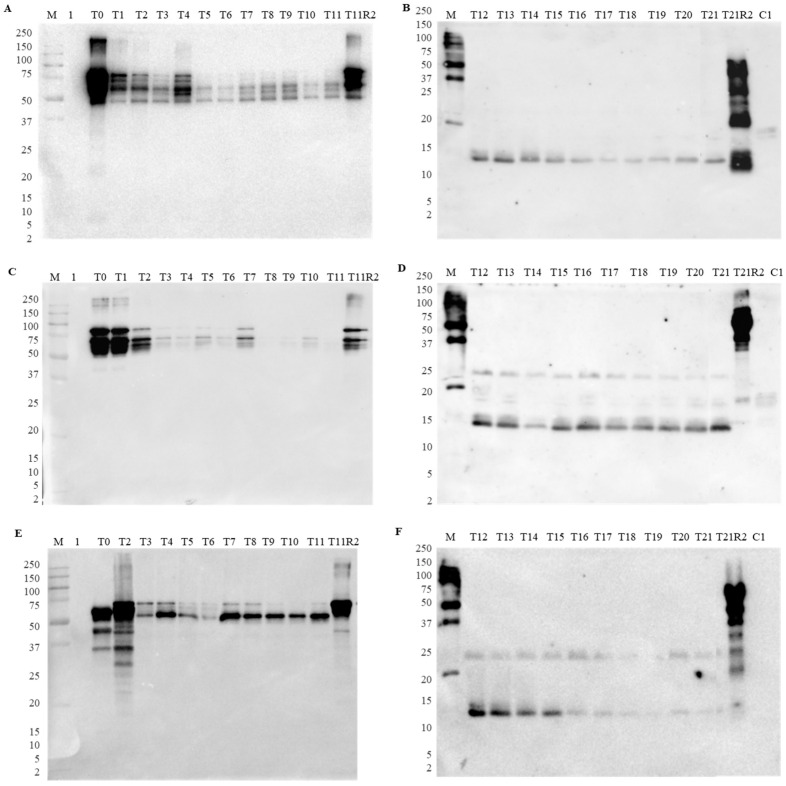
IgG binding capacity of γ-conglutin during salivary, gastric (**A**,**C**,**E**), and intestinal digestion with individual enzymes (trypsin/chymotrypsin) (**B**,**D**,**F**) of boiled pasta containing 35% of *L. luteus* (**A**,**B**), *L. angustifolius* (**C**,**D**), and *L. albus* (**E**,**F**). Legend: lane 1—pepsin; T0—0 min of salivary phase; T1—end of salivary phase; T2—0 min of gastric digestion; T3—5 min; T4—10 min; T5—15 min; T6—20 min; T7—30 min; T8—45 min; T9—60 min; T10—90 min; T11—120 min; T11R2—120 min of gastric digestion without enzymes; T12—0 min of intestinal digestion; T13—5 min; T14—10 min; T15—15 min; T16—20 min; T17—30 min; T18—45 min; T19—60 min; T20—90 min; T21—120 min; T21R2—120 min of intestinal digestion without enzymes; C1—negative control without boiled lupine pasta.

The time-dependent course of GI digestion was monitored by SDS-PAGE (Figure S1) and immunoblotting (Figure 2). Following the progress of the gastric phase, a continuous degradation of all protein bands was generally observed in all lupine species (Figure S1A,C,E). Similarly, immunoblotting results showed a decrease in the IgG binding of γ-conglutin, especially in *L. luteus* (Figure 2A) and *L. angustifolius* (Figure 2C). All bands below 50 kDa were degraded during gastric digestion, meaning that disulfide-linked subunits (mostly between 50 and 75 kDa) retained their immunoreactivity even after pepsin degradation, remaining observable until the end of the gastric phase (Lane T11, Figure 2A,E). However, in the case of *L. angustifolius*, IgG binding was almost completely abolished at the end of gastric digestion (lane T11, Figure 2C).

According to the harmonized digestion protocol from INFOGEST 2.0, different intestinal enzymes can be used, namely, pancreatin or trypsin/chymotrypsin. For that reason, in this work, two parallel intestinal digestions were performed to compare the use of pancreatin or individual enzymes (trypsin and chymotrypsin). Results obtained from the intestinal digestion using pancreatin showed that the anti-γ-conglutin antibody exhibited some unspecific reactivity with pancreatin in all pasta digestions with the appearance of several bands corresponding to its molecular weight. These results seem to be common when performing intestinal digestion of other legume species using pancreatin (unpublished results), which suggests a masking effect of the detection of target proteins, proving the interference of this enzyme in this specific matrix. Therefore, the results with pancreatin were inconclusive, and this phenomenon is currently being investigated in other matrices.

When using trypsin and chymotrypsin individually, a different protein pattern was obtained by both SDS-PAGE (Figure S1B,D,E) and immunoblotting (Figure 2B,D,F), providing a more trustful assessment of the results. In fact, the intestinal digestion of *L. luteus* and *L. albus* proteins showed a band around 15–18 kDa that lost intensity along time (Figure 2B,F). In the case of *L. angustifolius*, the same band seems to maintain the intensity during all intestinal digestion (Figure 2D). Additionally, a slight band at approximately 25–27 kDa was also detected in *L. angustifolius* and *L. albus* intestinal digestion, maintaining its immunoreactivity until the end of the digestion process (Figure 2D,F, lanes T21). These results confirm previous findings obtained by Czubinski, et al. [33] that showed the trypsin resistance of *L. angustifolius* γ-conglutin fraction, obtaining immunoreactive bands with the same molecular weight (28 kDa). These bands might correspond to digested peptides from monomer and subunits α- and β-γ-conglutin, which maintained their epitopes, even after being submitted to the proteolytic activity of trypsin and chymotrypsin [17,33]. Comparing the three species, it seems that *L. albus* proteins almost lost their immunoreactivity at the end of GI digestion, followed by *L. luteus*, and *L. angustifolius,* which had the highest band intensity, suggesting that the obtained peptides are differently affected by digestive enzymes.

ELISA data obtained at the end of the gastric digestion confirmed the immunoblotting results based on the significant reduction in immunoreactivity (98%) observed in all lupine species (Table 1). However, after the intestinal digestion, the protein was not detected, meaning that the system was not able to detect the residual IgG binding observed in immunoblotting (Figure 2).

In general, it seems that γ-conglutin submitted to a boiling process within a pasta matrix maintains a residual immunoreactivity even after GI digestion. It was already demonstrated that γ-conglutin has an intrinsic resistance to proteolysis, unless its native conformation is altered by the pH [34]. Trypsin- and chymotrypsin-cleavable peptide bonds (arginine, lysine, leucine, and aromatic residues) account for about 25% of total amino acids in the γ-conglutin sequence. Therefore, all these amino acid residues must be hardly accessible to pancreatic enzymes when γ-conglutin is in its native conformation. This protein has an extremely compact native conformation with six disulfide bridges that are responsible for its high stability, being less accessible to proteases. Therefore, the boiling process seems to partially affect the native conformation of the protein, which, combined with the relatively few trypsin peptide bond cleavage sites, maintains its resistance to GI digestive enzymes. Additionally, at physiological conditions (neutral pH), the occurrence of ionic interactions with flavonoids released from the oxidation of lupine seed polyphenols can also play a role during digestion with pancreatin, providing protection of γ-conglutin against enzymatic activity. Consequently, the digestion effectiveness was reduced.

## 4. Conclusions

In this work, the effect of thermal treatment combined with GI digestion on the immunoreactivity of γ-conglutin of three economically important lupine species was assessed. Other reports on food processing and digestibility studies applied to lupine proteins mainly rely on the use of lupine proteins concentrates or isolates, without verifying the involvement of a food matrix. In this case, model mixtures of homemade pasta containing lupine flour of each species provided a more realistic perspective of what effectively happens to a protein after being processed and submitted to GI digestion within a food matrix. Results clearly showed that γ-conglutin structure is differently affected depending on the lupine species, which can be due to the distinct degree of glycosylation in homologues proteins as it is a common PTM in these proteins. Similarly, the immunochemical assays performed using a specific anti-γ-conglutin antibody showed that the IgG binding of *L. luteus* decreased after thermal treatment, while it increased substantially in *L. albus*. The immunoreactivity of *L. angustifolius* remained unaffected.

For the first time, it was demonstrated that the use of isolated trypsin and chymotrypsin is a better option to study the GI digestion of lupine allergens than pancreatin, which seems to mask the efficient identification of digested peptides. The immunoreactivity after GI digestion of boiled pasta is also similarly affected by each species, with *L. albus* and *L. angustifolius* being more resistant to the proteolytic effect of intestinal enzymes. This resistance can be attributed to the compact structure of γ-conglutin, which hampers the cleavage sites of trypsin and chymotrypsin.

The application of severe food processing treatments followed by the study of the impact of GI digestion on lupine allergens is important for unveiling strategies to develop food formulations with reduced allergenicity. Herein, the combined results of thermal treatments with GI digestion suggest that the immunoreactivity of *L. luteus* was the most affected from the tested species, highlighting its potential use as a safer protein alternative for lupine-allergic patients.

## Figures and Tables

**Table 1:** Results obtained by indirect ELISA targeting γ-conglutin in raw and boiled pasta containing 35% of each lupine species and in digests after gastric (T11) and intestinal (T21) simulated digestion.

Species	Treatment	γ-Conglutin (mg/mL)	Coefficient of Variation (%)
*L. luteus*	Raw pasta	2.47 ± 0.11	4.54
	Boiled pasta	1.50 ± 0.24	15.6
	Boiling water	0.22 ± 0.02	9.12
	Gastric digestion (T11)	0.023 ± 0.001	6.22
	Intestinal digestion (T21)	n.d. *	-
*L. angustifolius*	Raw pasta	1.23 ± 0.14	11.2
	Boiled pasta	1.22 ± 0.06	5.07
	Boiling water	0.001 ± 0.000	24.8
	Gastric digestion (T11)	0.027 ± 0.001	4.73
	Intestinal digestion (T21)	n.d. *	-
*L. albus*	Raw pasta	0.023 ± 0.001	4.61
	Boiled pasta	1.17 ± 0.10	8.73
	Boiling water	0.0004 ± 0.0001	27.1
	Gastric digestion (T11)	0.024 ± 0.003	14.5
	Intestinal digestion (T21)	n.d. *	-

* n.d.—not detected.

## Data Availability

The original contributions presented in the study are included in the article/Supplementary Materials, further inquiries can be directed to the corresponding author.

## Supplementary Materials

The following supporting information can be downloaded at: https://www.mdpi.com/article/10.3390/foods13152330/s1, Figure S1: Protein profile by SDS-PAGE during gastric (A,C,E) and intestinal digestion with individual enzymes (B,D,F) of boiled pasta containing 35% of *L. luteus* (A,B), *L. angustifolius* (C,D), and *L. albus* (E,F) performed with pepsin.
