# Supplementary material for: γ-Conglutin Immunoreactivity Is Differently Affected by Thermal Treatment and Gastrointestinal Digestion in Lupine Species
Source: Foods. 2024 Jul 24;13(15):2330. doi: 10.3390/foods13152330 (PMC11312398; doi:10.3390/foods13152330)
Supplement: Supplementary file 1 [file foods-13-02330-s001.zip › foods-3121263-supplementary.pdf]

Supplementary material

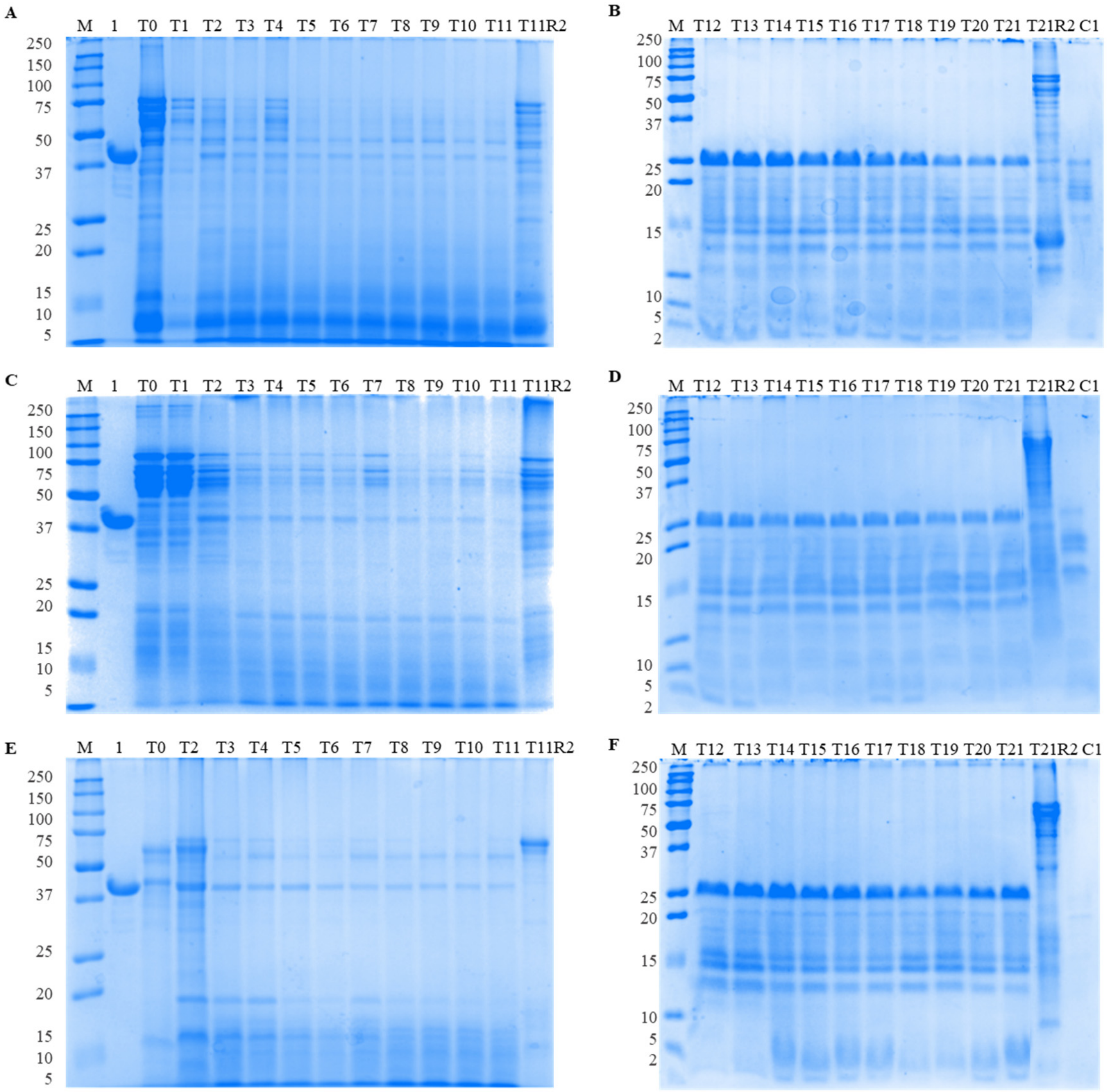

Figure S1 – Protein profile by SDS-PAGE during gastric (A, C, E) and intestinal digestion with individual enzymes (B, D, F) of boiled pasta containing 35% of *L. luteus* (A, B), *L. angustifolius* (C, D) and *L. albus* (E, F) performed with pepsin. Legend: lane 1, pepsin; T0, 0 min of salivary phase; T1, end of salivary phase; T2, 0 min of gastric digestion; T3, 5 min; T4, 10 min; T5, 15 min; T6, 20 min; T7, 30 min; T8, 45 min; T9, 60 min; T10, 90 min; T11, 120 min; T11R2, 120 min of gastric digestion without enzymes; T12, 0 min of intestinal digestion; T13, 5 min; T14, 10 min; T15, 15 min; T16, 20 min; T17, 30 min; T18, 45 min; T19, 60 min; T20, 90 min; T21, 120 min; T21R2, 120 min of intestinal digestion without enzymes; C1, negative control without boiled lupine pasta.
